# One-Step Sortase-Mediated Chemoenzymatic Semisynthesis of Deubiquitinase-Resistant Ub-Peptide Conjugates

**DOI:** 10.1021/acsomega.2c05652

**Published:** 2022-12-07

**Authors:** Avinash K. Singh, Sumit Murmu, Artur Krężel

**Affiliations:** † Department of Chemical Biology, Faculty of Biotechnology, University of Wrocław, F. Joliot-Curie 14a, 50-383 Wrocław, Poland; ‡ National Institute of Immunology, Aruna Asaf Ali Marg, New Delhi 110067, India

## Abstract

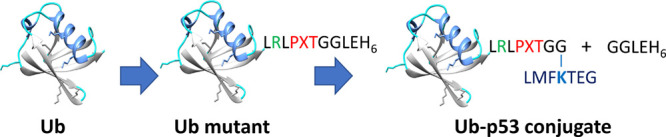

Post-translational modifications (PTMs) of proteins increase the functional diversity of the proteome and play crucial regulatory roles in cellular processes. Ubiquitination is a highly regulated and reversible PTM accomplished by a complex multistep process with the sequential action of several specific ubiquitinating (E1–E3) and deubiquitinating enzymes. The different types of ubiquitination (mono-, poly-mono-, and poly-) and the presence of several target sites in a single substrate add to its complexity, which makes the in vitro reconstitution of this ubiquitin (Ub) machinery a quite cumbersome process. Defects in components of the ubiquitination process also contribute to disease pathogenesis, especially cancer and neurodegeneration. This makes them of interest as potential therapeutic targets. Therefore, the development of efficient and reliable methods that will generate a highly homogeneous ubiquitinated peptide and protein conjugate is a topical subject area of research. In this report, we describe the development of a simple and efficient in vitro sortase-mediated chemoenzymatic strategy for semisynthesis of defined and homogeneous ubiquitin conjugates with more than 90% yield. This was achieved by engineering a sortase recognition motif in the dynamic C-terminus of ubiquitin and its conjugation to an isopeptide-linked di-Gly appended peptide LMFK(ε-GG)TEG corresponding to the ubiquitination site residues _383_LMFKTEG_389_ of p53. The defined and homogeneous ubiquitin conjugates were also weighed for their recognition propensity by deubiquitinating enzymes. This facile semisynthesis of ubiquitin conjugates establishes a simple one-step sortase-mediated chemoenzymatic route for the synthesis of homogeneous and defined isopeptide-linked polypeptides and will help in understanding the complexity of the ubiquitination machinery as well as designing isopeptide drugs and therapeutics.

## Introduction

Ubiquitination is a highly regulated, reversible covalent protein post-translational modification (PTM) in eukaryotes, in which the C-terminal glycine of ubiquitin (Ub) molecules is conjugated via isopeptide linkage to the ε-amine of a lysine residue of the target proteins.^1,2^ Ub is a highly conserved 8.5-kDa, 76-residue-long polypeptide (Table S1), whose role in intracellular protein degradation has been well described.^3−6^ Three types of ubiquitination can be found in a cell; monoubiquitination, in which a single ubiquitin is attached to the ε-Lys of the target proteins; multimonoubiquitination, in which the monoubiquitination process is repeated to attach Ub molecules to multiple lysine residues; and polyubiquitination, in which multiple Ub molecules are conjugated on a single lysine of the substrate.^3,14,17−23^ Ubiquitination of proteins can mark them for degradation via the proteasome, alter their cellular location, affect activity, and promote or prevent protein interactions. The fate of a ubiquitinated protein in a cell depends on the site and the type of ubiquitination involved. Polyubiquitination leads to different responses based on the position of the isopeptide bond and the number of ubiquitin molecules attached to the target protein.^7^ Only polyubiquitination on defined lysines, mostly on K48 and K29, is related to degradation by the proteasome (referred to as the “molecular kiss of death”), while other polyubiquitinations (e.g., on K63, K11, K6, and M1) and monoubiquitinations regulate other processes, such as endocytic trafficking, inflammation, translation, and DNA repair.^7−9,12,13^ Defects in machinery components of the ubiquitination process can contribute to disease pathogenesis, especially cancer and neurodegeneration.^14−16^ This makes them of interest as potential therapeutic targets. Ub-based probes and assay reagents are important as they help to provide insight into enzymatic activity and substrate recognition.^17^ The enzymes involved in this process are very specific and many of them still need to be characterized.^10,18^ The different types of ubiquitination and the presence of several target sites in a single substrate further add to its complexity.^7,9−11^ Due to these complications, the in vitro reconstitution of this ubiquitin machinery is quite cumbersome. Therefore, the development of efficient and reliable methods that will generate highly homogeneous predefined ubiquitinated peptide and protein conjugates is a topical subject of research.

Looking at the future possibility of developing novel potential therapeutics based on PTMs, in particular ubiquitination, several approaches have been developed for the synthesis of site-specific ubiquitin conjugates.^18−25^ All these approaches are based on a central theme requiring the synthesis of defined peptide fragments derivatized with reactive functionalities for their linkage through a native or a surrogate amide bond. The first approach for chemical ubiquitination utilized a photolytically removable ligation auxiliary for the ubiquitination of synthetic peptides.^19^ Inspired by this work, the γ- and δ-thiolysine-mediated ubiquitination methods were later developed.^20,21^ In these approaches, a site-specific ligation is enabled by the mercaptolysine’s thiol between the thioester of Ub and the thiol of substrate proteins followed by a desulfurization step resulting in ubiquitinated conjugates with native isopeptide bonds. In another approach, the rapid assembly of Ub using a premade 76 amino acid residue long isoUb synthon having a native isopeptide bond was achieved.^22^ This method was used along with hydrazide-based native chemical ligation to synthesize K11/K48-branched Hexa-Ub. Recently, K6- and K29-linked diubiquitins were synthesized through a genetically encoded orthogonal protection and activated ligation (GOPAL) approach combining genetic-code expansion, intein chemistry, and chemoselective ligation.^18^ This method was modified by replacing the benzyloxycarbonyl (Cbz) with the allyloxycarbonyl (Alloc) group, which can be removed under milder conditions, for the synthesis of more complicated oligo-ubiquitins.^23^ Recently, with the discovery and characterization of various ligases, i.e., sortase, asparaginyl endopeptidase (AEP), butelase, and Ubc9, chemoenzymatic synthesis of Ub conjugates has gained attention.^24,26−28^ Pawale et al. used sortase-mediated ligation (SML) for the semisynthesis of SUMO conjugates.^26^ Lang and co-workers developed another strategy to ubiquitinate proteins in vivo in an inducible fashion by combining sortase-mediated transpeptidation with genetic-code expansion and biorthogonal Staudinger reduction.^24^ Another chemoenzymatic method known as lysine acylation using conjugating enzymes (LACE) has also been reported for site-specific modification of folded proteins at internal lysine residues in a programmable manner.^27^ This method utilizes the intrinsic sequence specificity of Ubc9, an E2-SUMO-conjugating enzyme, for in vitro conjugation of wildtype Ub and ISG15 to recombinant proteins. In a very recent combinatorial approach, AEP in combination with genetic-code expansion was used for the synthesis of site-specific Ub and SUMO conjugates in vivo in an inducible fashion.^28^

Sortases are cysteine transpeptidase enzymes present in Gram-positive bacteria and are responsible for the covalent anchoring of surface proteins on the bacterial cell surface.^29−32^ The sortase A from *Staphylococcus aureus* (SaSrtA) is a prototype sortase and has emerged as a versatile tool for chemical biologists in manipulating proteins and peptides. It recognizes an LPXTG pentapeptide motif in the C-terminal of sortase substrates, cleaves the T–G peptide bond, and forms an acyl-enzyme intermediate, which is resolved by nucleophilic attack of an aminoglycine.^33,34^ Another class E sortase from *Streptomyces avermitilis* (SavSrtE) has been reported to recognize LPXTG as well as LAXTG pentapeptide motifs as substrates.^35^ SML has been utilized previously for the semisynthesis of defined engineered SUMO (eSUMO) conjugates, which involved the conjugation of eSUMO proteins to a prefabricated isopeptide-linked SUMO target peptide.^26^ The resultant SUMO conjugates were recognized by desumoylating enzymes of the sentrin-specific protease (SENP) family and served as their bona fide substrates. Since the ubiquitin conjugates prepared with genetic-code expansion fail to be recognized by deubiquitinating (DUB) enzymes but the SUMO conjugates generated by SML are recognized by desumoylating enzymes, it was of interest to explore whether Ub conjugates prepared by a similar SML strategy would be recognized by deubiquitinating enzymes as the ones prepared.

The present report describes the engineering of a sortase recognition motif in ubiquitin to develop a one-step sortase-mediated in vitro chemoenzymatic strategy for semisynthesis of defined Ub conjugates and assessment of their recognition propensity by deubiquitinating enzymes. For this, the C-terminal region containing a highly flexible unstructured conserved motif (LR_72_LR_74_GG) was converted into a sortase pentapeptide recognition motif by point mutation of R_72_ and R_74_ to Pro and Thr, respectively, creating LPLTGG. Alternatively, PXT substitution/insertion for R_74_ in the C-terminal “L_71_RLR_74_GG” sequence will also be engineered to get an extended Ub containing the LRLPXTGG sortase recognition motif. The engineered Ubs will be conjugated to a prefabricated isopeptide-linked di-Gly appended Ub target peptide derived from p53 protein.

## Experimental Section

### Materials

Bacterial plasmid pHA-ubiquitin (pcDNA3) was a gift from Yeh and co-workers (Addgene plasmid #18712).^36^ The oligonucleotides were custom synthesized from Sigma. The pET-23b(+) vector was obtained from Novagen. PCR reagents were purchased from Thermo Fisher Scientific, USA. Restriction enzymes and ligation kits were procured from New England Biolabs, U.K. *E.coli* DH-5α and XL1-Blue Competent Cells were procured from Thermo Fisher Scientific, USA. DNA isolation and gel extraction kit were purchased from Qiagen, USA. A QuikChange II XL Site-Directed Mutagenesis Kit was purchased from Agilent. LB medium was obtained from Biobasic, Canada. Tryptone, yeast extract, and agar were purchased from Pronadisa, Spain. Ampicillin and IPTG were obtained from Sigma. BL21-Gold (DE3) cells were obtained from Agilent. Ni-NTA beads and polypropylene columns were purchased from Qiagen, USA. The protein marker was purchased from Thermo Fisher Scientific. Amicon was purchased from Millipore. The PD-10 desalting column was purchased from GE Healthcare, U.K. Fluromethoxycarbonyl (Fmoc) amino acids and resins were purchased from Novabiochem. Peptide synthesis reagents HBTU, DIPEA, piperidine, DMF, hydrazine, and TFA were obtained from Applied Biosystems or Sigma. HPLC-grade acetonitrile (ACN) was obtained from Merck, and diethyl ether was obtained from Spectrochem, India. Deubiquitinating enzymes were purchased from Boston Biochem, USA. All other chemicals were obtained from Sigma-Aldrich unless otherwise indicated.

### ESI and Matrix-Assisted Laser Desorption/Ionization (MALDI)-TOF MS

The mass measurements were made by MALDI using a TOF 4800 or 5800 analyzer (AB SCIEX, USA) or electrospray on a Micromass LCT analyzer (Waters, USA).

### Subcloning and Site-Directed Mutagenesis

*E. coli* stab culture containing pHA-ubiquitin (pcDNA3) was subcultured in LB medium with 100 μg/mL ampicillin. The plasmid was isolated using Qiagen’s plasmid miniprep alkaline lysis protocol. The plasmid DNA was used as a template to PCR amplify the Ub gene using oligonucleotides nested with NdeI (forward) and XhoI (reverse) restriction sites. The amplicon obtained was digested with NdeI and XhoI restriction enzymes and subcloned into the pET-23b(+) vector under the same sites. The resultant clone was used as a template in site-directed mutagenesis for introducing the LPXTG sortase recognition motif into the highly flexible unstructured C-terminal motif (LRLRGG) of the Ub protein.

### Expression and Purification

The clones of SavSrtE and SaSrtA, namely SavSrtEΔ50-pET28b (N-term His-tag) and SaSrtAΔ59-pET23b (C-term His-tag), were available in our laboratory.^35,37^ The plasmids containing SavSrtEΔ50, SaSrtAΔ59, WT Ub, and mutant Ubs were transformed in BL21-Gold (DE3) competent cells for protein expression. The primary culture was grown overnight at 37 °C in LB medium containing 100 μg/mL ampicillin or 50 μg/mL kanamycin. The overnight grown culture was used for the inoculation of secondary culture, which was grown at 37 °C until A_600_ reached 0.4–0.6. This was followed by induction with 0.2 mM IPTG for protein expression for 3 h at 37 °C (SaSrtA and Ubs) and 12 h at 25 °C for SavSrtE. The cells were harvested by centrifugation (5000 rpm, 15 min, and 4 °C), resuspended in resuspension buffer (10 mM Tris pH 7, 40 mM NaCl, and 10 mM imidazole), and lysed by sonication, and the cell lysate was clarified by centrifugation (10,000 rpm, 30 min, and 4 °C). The supernatant was allowed to bind with Ni-NTA beads preequilibrated in resuspension buffer at 4 °C for 4 h. The beads were subsequently washed with wash buffer (10 mM Tris pH 7, 500 mM NaCl, and 30 mM imidazole). The protein was eluted with elution buffer (10 mM Tris pH 7, 40 mM NaCl, and 350 mM imidazole), concentrated in Amicon-Ultra concentrator (3/10 kDa MWCO), and desalted on a PD-10 column. The purity of each of the purified protein samples was analyzed by SDS-PAGE and RP-HPLC. Molecular masses of the proteins were confirmed by MALDI-TOF MS or ESI-MS (Table S2).

### Solid-Phase Peptide Synthesis

The peptide substrates were synthesized at the 0.1 or 0.25 mmole scale on an automated (433A, ABI) or semiautomated (Endeavor 90, Aapptec) solid-phase synthesizer employing 9-Fmoc SPPS chemistry.^38^ Wang resin preloaded with the desired amino acid was used as the starting material for synthesis. Fmoc-protected amino acids were coupled on the deprotected resin using HBTU/DIPEA coupling. The synthesis began with deprotection of the Fmoc group on the amino acid preloaded on the resin in the presence of 20% piperidine/80% DMF. The desired sequence was assembled through repeated cycles of deprotection and coupling.^39^ The N-terminal Leu of LMFKTEG peptide encompassing the Ub target site of p53 corresponding to residues 383–389 was protected with a *tert*-butoxycarbonyl (Boc) group, and the Lys side chain was protected with 1-(4,4-dimethyl-2,6-dioxacyclohexylohexylidene)-ethyl (Dde group), orthogonal to the Boc and Fmoc group (Fmoc-Lys(Dde)-OH), to facilitate the elaboration of di-Gly branching on Lys. After completion of the linear peptide assembly, the Dde group was deprotected using 5% hydrazine prepared in DMF. Boc-Gly-Gly was then coupled to ε-amine of lysine. Peptide resins obtained after completion of synthesis were washed thoroughly with DMF and DCM and then vacuum dried in a desiccator. A mixture of 95% TFA and 5% water was used for removal of the side-chain protections and cleavage of the peptides from vacuum dried resin. 2.5% EDT was included in the mixture when the peptide sequence contained an amino acid side chain having a trityl (Trt) protecting group. The cleavage reaction was carried out for about 2–2.5 h at room temperature.^40^ The cleaved peptides were precipitated in ice-cold diethyl ether followed by extraction in water and lyophilization. The crude peptides were purified by RP-HPLC using a preparative C-18 column (Phenomenex, 100 Å, 10 μm, and 30 × 250 mm) employing a linear gradient of 8–72% ACN in 0.1% TFA (flow rate: 30 mL/min). Purified peptides were lyophilized, dissolved in a solvent containing 50% ACN and 0.1% formic acid, and subjected to MALDI-TOF or ESI-MS for mass determination (Table S3).

### Sortase-Mediated Transpeptidation Reaction

The peptide conjugation reactions were carried out using 0.5 mM Ub mutant, 1 mM LMFK(ε-GG)TEG or GGGKY, and SaSrtA (50 μM)/SavSrtE (100 μM) in SaSrtA reaction buffer (150 mM NaCl, 50 mM Tris pH 7.5, 5 mM CaCl_2_, and 2 mM β-mercaptoethanol) at 37 °C or SavSrtE reaction buffer (150 mM NaCl, 50 mM Tris pH 7.0, and 2 mM β-mercaptoethanol) at 20 °C. The reaction was analyzed on a C-18 RP-HPLC column (Phenomenex, Luna 5 μm, 100 Å, and 4.6 × 250 mm) using a linear gradient of ACN (8–72% in 130 min) and 0.1% TFA. The product peak was collected, lyophilized, and analyzed by mass spectrometry (Table S4).

### Deubiquitination Assay

The deubiquitinating enzymes were activated by preincubating them with 10 mM dithiothreitol (DTT) prior to the deubiquitination reaction, and their activity was analyzed on their standard substrates (Figure S7). Purified peptide conjugate (0.5 mM) was taken in water and incubated with 1 μM of deubiquitinating enzymes in a buffer containing 150 mM NaCl, 50 mM Tris pH 8.0, and 5 mM β-mercaptoethanol at 37 °C for 1 h. The reaction was analyzed on a C18 RP-HPLC column using a linear gradient of ACN (4–72% in 130 min) and 0.1% TFA. The product peak was collected, lyophilized, and analyzed by mass spectrometry (Table S4).

## Results

To obtain Ub conjugates by SML in the first part of our work, we overproduced and purified two types of sortases, SavSrtE, and SaSrtA. These cysteine transpeptidases (SavSrtE and SaSrtA) are housekeeping enzymes from *S. avermitilis* and *S. aureus*, respectively. They both recognize an LPXTG pentapeptide motif as their substrates. Sortase A has been used comprehensively as a tool for protein engineering, protein–protein, protein–peptide, and peptide–peptide ligation, but sortase E has been used here for the first time as a similar tool. With both sortases in hand, the substrates of the SML, peptides, and ubiquitin variants were obtained chemically using SPPS peptide synthesis and bacterial production, respectively. Using a few different strategies, several Ub proteins with various sortase-recognizing sequences were produced (WT and mutants). Then, by applying two different sortases, two types of peptides, and a series of Ub variants, examination of the one-step chemoenzymatic semisynthesis of Ub conjugates was performed. The conjugates that were obtained in great purity and high yield were finally examined regarding their resistance against deubiquitinating enzymes.

### Cloning, Mutagenesis, and Expression

Both of the sortases, SavSrtEΔ50 and SaSrtAΔ59, were expressed in *E. coli* with a hexa-His-tag at their N-terminus (pET-28b) and C-terminus (pET-23b), respectively. Expression of both proteins was induced with IPTG at 20 and 37 °C, respectively, as it was previously optimized.^26,35^ Overproduced proteins were bound to the Ni^2+^-loaded NTA column during purification and were eluted with 350 mM imidazole. Collected enzyme fractions were concentrated using 10 kDa MWCO Amicon-Ultra and purified by SEC using PD-10 columns. 10 and 30 mg/L of SavSrtEΔ50 and SaSrtAΔ59, respectively, were purified with more than 90% purity (Figure S1). Identity of both proteins was confirmed by mass spectrometry, while activity was tested using the standard short peptide substrates YNLPETGA and GGGKY as reported earlier (data not shown).^35^

**Figure 1:**
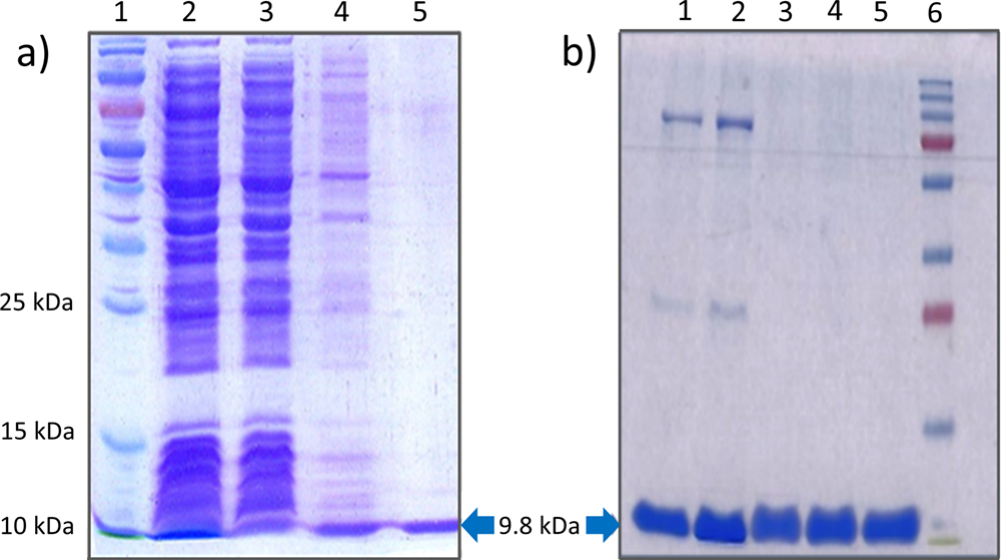
Purification of WT Ub and Ub mutants. (a) Purification profile of WT Ub protein. Lane 1: protein ladder, Lane 2: lysate, Lane 3: flow-through, Lane 4: wash, and Lane 5: eluted Ub; and (b) purification profile of Ub mutants. Lane 1: Ub-LPLTGG, Lane 2: Ub-LPNTGG, Lane 3: Ub-LRLPNTGG, Lane 4: Ub-LRLPETGG, Lane 5: Ub-LRLPLTGG, and Lane 6: protein ladder.

To obtain Ub conjugates, the sortase recognition motif LPXTG containing LPLTGG was generated at the C-terminal end of ubiquitin, which offers a native LRLRGG sequence (Table S1). The HA-ubiquitin insert in pHA-ubiquitin (pcDNA3) plasmid is from the UBB gene and contains a single ubiquitin coding sequence of 228 bp. The PCR amplified gene of Ub was visualized on agarose gel and was subcloned into the pET-23b vector (as described in the Materials section). Ub-pET23b plasmid DNA encoding Ub was used as a template, and a modified Ub variant was made by site-directed mutagenesis of the R_72_ and R_74_ residues to Pro_72_ and Thr_74_, respectively, using SDM primers, whose sequences are listed in Supporting Information (Table S5). After mutagenesis, the C-terminal end of ubiquitin (LR_72_LR_74_GG) became LP_72_LT_74_GG, which is a potential sortase recognition motif (LPXTG). An additional mutant with variation at the X position, comprising the LPNTG sortase recognition motif, was also made by site-directed mutagenesis of Leu_73_ to Asn_73_. However, in test sortase assays using GGGKY peptide as a nucleophile substrate, both the mutants failed to produce any transpeptidation product. Therefore, other three ubiquitin mutants, namely Ub-LRLPNTGG, Ub-LRLPETGG, and Ub-LRLPLTGG, were generated by substitution/insertion of PNT/PET/PLT for R_74_ in the C-terminal “L_71_RLR_74_GG” sequence (see Supporting Information for details). Finally, Ub-WT and Ub mutants cloned in the pET23b vector were expressed with hexa-His-tag (from the plasmid backbone) at their C-terminus. Protein expression was induced by 0.2 mM IPTG at 37 °C for 3 h and purified using Ni-NTA affinity purification (Figure 1a), and their purity was checked by SDS-PAGE and RP-HPLC. The SDS-PAGE profile of purified Ub mutants (Figure 1b) showed a single band that migrated in accord with the molecular weight, indicating homogeneity of the protein preparation. All Ub mutants were produced with high purity (Figure S2) and yield, which depending on the Ub protein varied from 30 to 40 mg/L. The mass of the mutant proteins was determined by MALDI-TOF MS and was in accord with the expected mass (Table S2). The SavSrtE and SaSrtA enzymes were also purified using Ni-NTA affinity purification followed by SEC on a PD-10 column. The purity of the enzymes was analyzed by SDS-PAGE (Figure S1) and RP-HPLC, and their identity was confirmed using mass spectrometry (Table S2). All the purified proteins were concentrated using Amicon-Ultra concentrators, and their aliquots were stored at −20 °C.

### Synthesis and Characterization of Amino-Nucleophile Peptides

**Figure 2:**
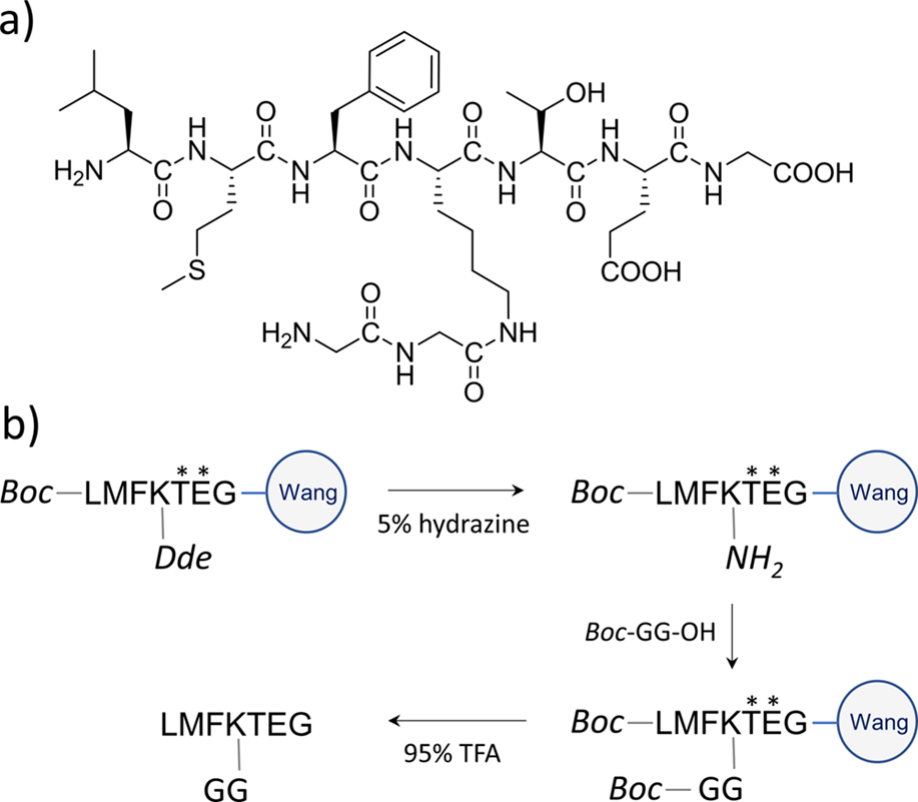
Synthesis of LMFK(ε-GG)TEG peptide for the sortase-mediated ligation with Ub mutants. (a) Structure of LMFK(ε-GG)TEG. (b) Particular steps of peptide synthesis. Italic letters represent protective groups important for orthogonal synthesis. Asterisks highlight amino acid residues, whose sidechains were protected and are presented for clarity.

To explore the feasibility of SML of Ub-LRLPXTGG mutant proteins, the standard amine donor peptide GGGKY was synthesized using standard SPPS chemistry described in Materials section (Figure S3). The peptide was obtained in a high yield since its synthesis was based on a single polypeptide chain elongation. GGGKY peptide was used for testing the activity of SaSrtA and SavSrtE as well as the recognition propensity of modified Ubs by these sortases. The second peptide containing the ubiquitination motif LMFK(ε-GG)TEG corresponds to the ubiquitination site residues _383_LMFKTEG_389_ of p53 elaborated with Gly-Gly residues at ε-NH_2_ of K_386_ (Figure 2a). Its synthesis is based on the orthogonal synthesis of the main linear chain LMFKTEG with subsequent modification of its lysine residue at its ε-amine. An orthogonal approach was achieved through the use of a 2,6-dioxacyclohexylohexylidene)-ethyl (Dde) group at K_386_, which was selectively removed by 5% hydrazine without removing other groups (Figure 2b). This peptide was obtained in a slightly lower amount as compared to GGGKY. Both amino-nucleophile peptides were purified by RP-HPLC to 99 and 95% purity, respectively, and their identity was confirmed by MALDI-TOF mass spectrometry (Table S3).

### Sortase-Mediated Conjugation of Recombinant Ubiquitins with GGGKY

**Figure 3:**
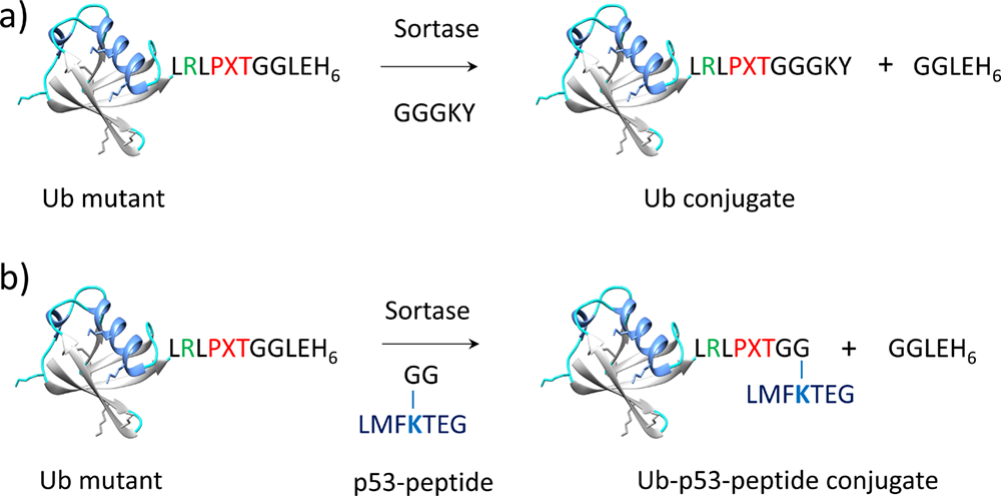
Schematic representation of sortase-mediated semisynthesis of defined ubiquitin conjugate. (a) Schematic representation of reaction between Ub mutants and GGGKY. (b) Schematic representation of reaction between Ub mutants and p53-peptide.

To explore whether modified Ub mutants (Ub-LRLPNTGG, Ub-LRLPETGG, and Ub-LRLPLTGG) were recognized by SaSrtA and SavSrtE as a substrate or not, SML of modified Ubs was first carried out with standard GGGKY substrate in SaSrtA reaction buffer (150 mM NaCl, 50 mM Tris pH 7.5, 5 mM CaCl_2_, and 2 mM β-ME) at 37 °C or SavSrtE reaction buffer (150 mM NaCl, 50 mM Tris pH 7.0, and 2 mM β-ME) at 20 °C (Figure 3a). Separate individual controls for all the reactants were used. After 6 h, the reaction was quenched with 0.1% aqueous TFA and analyzed on a C-18 RP-HPLC column using a linear gradient of ACN (8–72% in 130 min). The HPLC profile of the transpeptidation reaction between Ub-LRLPNTGG and GGGKY confirmed the formation of the ubiquitinated product Ub-LRLPNTGGGKY and GGLEH_6_ along with the respective peaks for GGGKY, SaSrtA (or SavSrtE), and Ub-LPNTGG (Figure S4). The area under the respective peaks was calculated, and the product formation was measured as percentage conversion of the concentration-limiting substrate Ub to the ligated product. The product conversion obtained with SaSrtA was 85% while that with SavSrtE was 22%, indicating that both SaSrtA and SavSrtE catalyzed reactions worked on the recombinant Ub-LRLPNTGG and GGGKY peptide, but the former was more efficient. Similarly, the HPLC profile of the reaction mixture of Ub-LRLPETGG and GGGKY produced the ubiquitinated product Ub-LRLPETGGGKY and GGLEH_6_ along with the respective peaks for GGGKY, SaSrtA (or SavSrtE), and Ub-LPETGG (Figure S5). The conversion obtained with SaSrtA was ∼61% in 6 h, while with SavSrtE it was found to be ∼20% in 6 h. Curiously, RP-HPLC analysis of the transpeptidation reaction between Ub-LRLPLTGG and GGGKY in the presence of SaSrtA or SavSrtE did not yield any transpeptidation product (Figure S6), indicating that Ub-LRLPLTGG was not recognized by sortase, possibly due to some altered conformational feature of the C-terminus.

### Sortase-Mediated Conjugation of Recombinant Ubiquitins with p53-Peptide Construct

**Figure 4:**
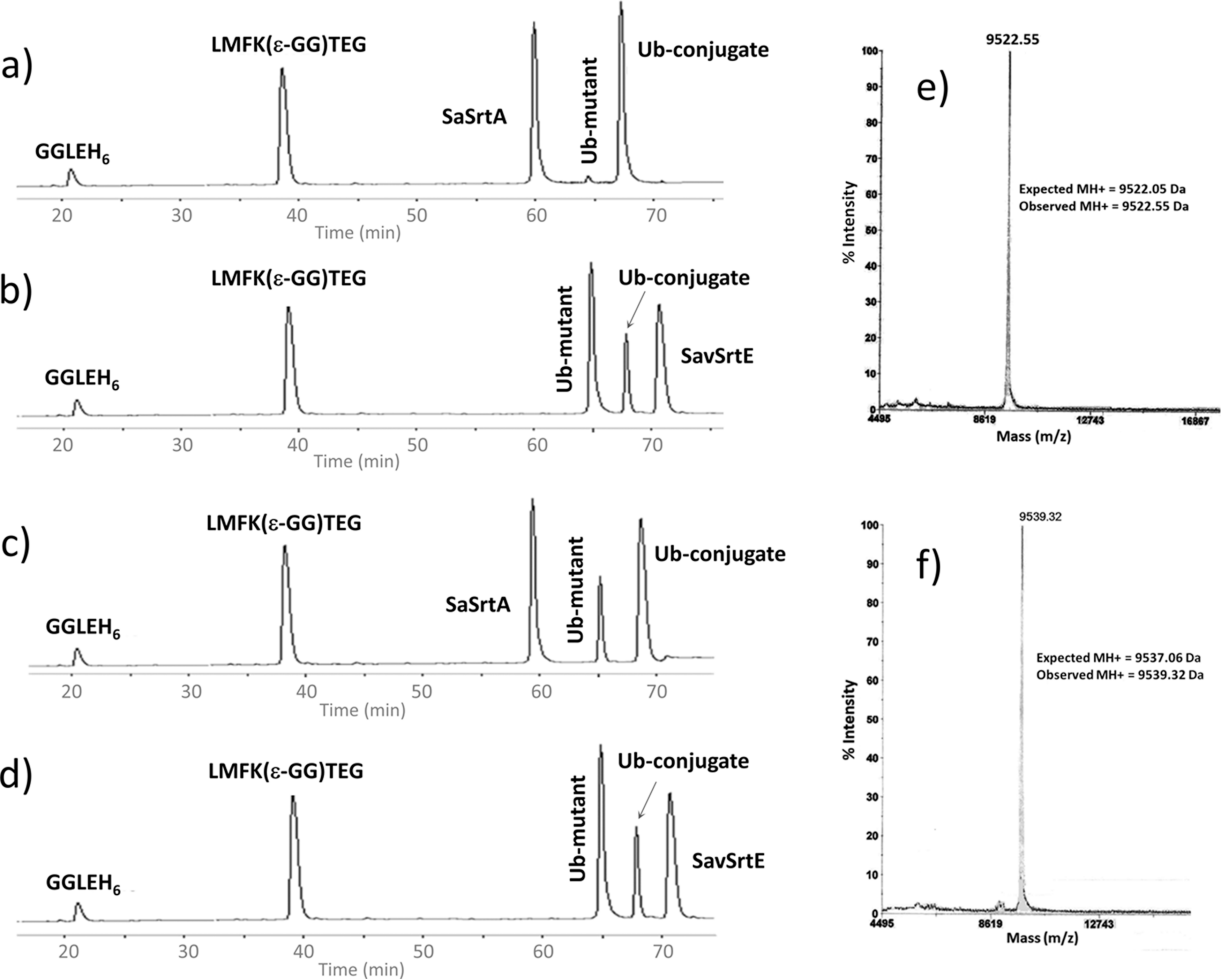
Sortase-mediated synthesis of Ub-p53 peptide conjugates. Reaction was analyzed on a C-18 RP-HPLC column with a linear gradient of 8–72% ACN in 130 min, and absorbance was recorded at 210 nm. (a) RP-HPLC analysis of the reaction between Ub-LRLPNTGG and LMFK(ε-GG)TEG peptide with SaSrtA: the reaction was carried out using 0.5 mM Ub-LRLPNTGG, 1 mM LMFK(ε-GG)TEG, and 50 μM of SaSrtA. (b) RP-HPLC analysis of the reaction between Ub-LRLPNTGG and LMFK(ε-GG)TEG peptide with SavSrtE: the reaction was carried out using 0.5 mM Ub-LRLPNTGG, 1 mM LMFK(ε-GG)TEG, and 100 μM of SavSrtE. (c) RP-HPLC analysis of the reaction between Ub-LRLPETGG and LMFK(ε-GG)TEG peptide with SaSrtA: the reaction was carried out using 0.5 mM Ub-LRLPETGG, 1 mM LMFK(ε-GG)TEG, and 50 μM of SaSrtA. (d) RP-HPLC analysis of the reaction between Ub-LRLPETGG and LMFK(ε-GG)TEG peptide with SavSrtE: the reaction was carried out using 0.5 mM Ub-LRLPETGG, 1 mM LMFK(ε-GG)TEG, and 100 μM of SavSrtE. (e) The observed *m/z* for Ub-LPNTLMFK(ε-GG)TEG was 9522.55 Da and was in correspondence with the expected *m/z* of 9522.05 Da. (f) The observed *m/z* for Ub-LPETLMFK(ε-GG)TEG was 9539.32 Da against the expected *m/z* of 9537.06 Da.

The p53 protein, also known as the guardian of the genome, is a 393 residue (in humans) long penta-domain protein that has been extensively studied for its regulatory and functional roles. The p53 tumor suppressor protein is a transcription factor that prevents oncogenic progression by activating the expression of apoptosis and cell-cycle arrest genes in stressed cells. The p53 protein has been reported to be post-translationally modified, and its stability is tightly regulated by polyubiquitination and degradation by the 26S proteasome. There are at least 14 potential ubiquitination sites in the p53 protein, and we have used one of these ubiquitination sites, Lys_386_ (LMFK_386_TEG), for synthesizing Ub-p53 conjugates. The sortase-mediated transpeptidation of the engineered Ub protein Ub-LRLPNTGG was carried out with LMFK(ε-GG)TEG in the presence of SaSrtA or SavSrtE for 10 h as per the reaction conditions described earlier (Figure 3b). Separate individual controls for all the reactants were used. Subsequently, RP-HPLC analysis of the reaction mixture confirmed the formation of the ubiquitinated product Ub-LRLPNTLMFK(ε-GG)TEG with both the sortase enzymes (Figure 4a,b). The identity of these conjugates was confirmed by MALDI-TOF MS. The percent product conversion was calculated from the respective peak areas as percentage conversion of the concentration-limiting substrate Ub to the ligated product. With SaSrtA (Figure 4a), more than 90% of product formation was observed for Ub-LPNTGG and LMFK(ε-GG)TEG, while with SavSrtE (Figure 4b), the product formation observed was ∼35%. This was as per expectations as SaSrtA has high catalytic efficiency, specificity, and selectivity for LPXTG recognition motifs as compared to SavSrtE, which recognizes LPXTG as a recognition motif but has a preference for LAXTG motif.

Similarly, the RP-HPLC analysis of the reaction mixture for the sortase-mediated transpeptidation reaction between engineered Ub protein Ub-LRLPETGG and LMFK(ε-GG)TEG in the presence of SaSrtA or SavSrtE for 10 h confirmed the formation of the ubiquitinated product Ub-LRLPETLMFK(ε-GG)TEG with both the enzymes (Figure 4c,d). For Ub-LRLPETGG, the product formation calculated from the peak area was ∼70% with SaSrtA (Figure 4c), while with SavSrtE (Figure 4d), it was found to be about ∼35%.

The recognition of engineered LRLPXTGG motif in engineered Ub by sortase and its successful conjugation with GGGKY and di-Gly appended p53 peptide illustrate the acquiescence of the dynamic and conserved C-terminal motif LRLRGG of Ub as well as the di-Gly appended p53 peptide for sortase-mediated transpeptidation. The recognition of di-Gly appended p53 peptide by sortase shows that the introduction of di-Gly at the ubiquitination site residues by SPPS is another means in addition to genetic-code expansion, which is useful for obtaining sortase-mediated Ub-peptide conjugates.

### Action of USP-7 and UCHL-3 on Ub-p53 Isopeptide Conjugates

Deubiquitinating enzymes (DUBs) are thiol proteases that catalyze the removal of Ub by hydrolysis of thioesters or isopeptide bonds of the Ub-conjugated substrate. This process is called deubiquitination and is responsible for the salvaging, processing, and editing of ubiquitin. It prevents the degradation of free Ub molecules from the small intracellular nucleophiles glutathione and polyamines and also serves as a proofreading mechanism by rescuing inappropriately targeted proteins from degradation.^41,42^

**Figure 5:**
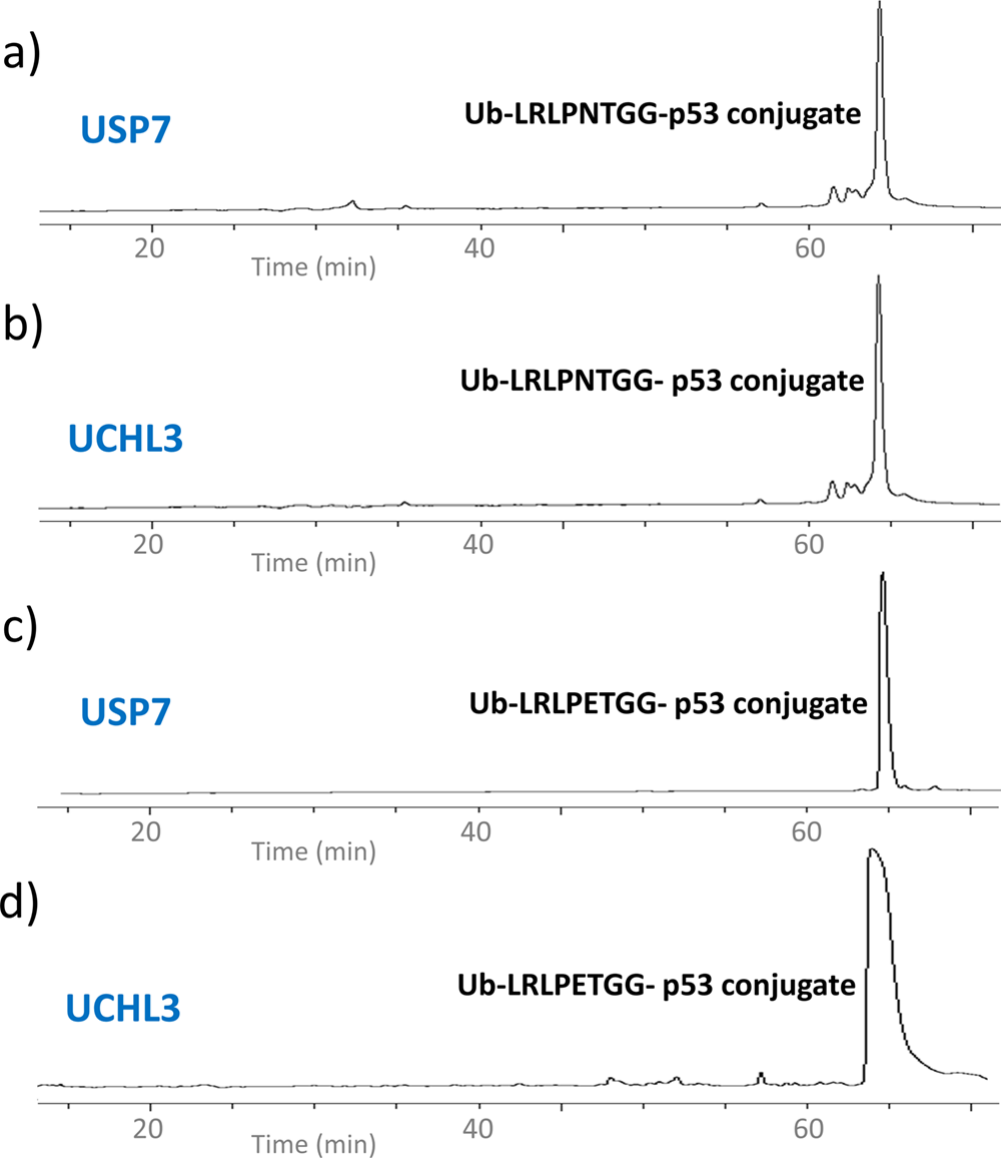
Deubiquitination of Ub-p53-peptide conjugates. 0.5 mM Ub-p53-peptide conjugate was incubated with 1 μM of DUB at 37 °C for 1 h and was analyzed at 210 nm on a C-18 RP-HPLC column with a linear gradient of 4–72% ACN in 130 min. (a) RP-HPLC analysis of the reaction between Ub-LRLPNTLMFK(ε-GG)TEG and USP7; (b) RP-HPLC analysis of the reaction between Ub-LRLPNTLMFK(ε-GG)TEG and UCHL3; (c) RP-HPLC analysis of the reaction between Ub-LRLPETLMFK(ε-GG)TEG and USP7; and (d) RP-HPLC analysis of the reaction between Ub-LRLPETLMFK(ε-GG)TEG and UCHL3.

To test the processing of the Ub-p53-peptide conjugates by deubiquitinating (DUBs) enzymes, purified Ub-LPNTLMFK(ε-GG)TEG and Ub-LPETLMFK(ε-GG)TEG conjugates were incubated with the deubiquitinating enzyme USP-7. This deubiquitinating enzyme is a member of the peptidase C19 family and is primarily involved in the deubiquitination of p53. However, no new hydrolysis peak was observed in the RP-HPLC analysis profile of the USP-7 treated samples of either conjugate (Ub-LRLPNTLMFK(ε-GG)TEG or Ub-LRLPETLMFK(ε-GG)TEG), indicating that both conjugates were resistant to hydrolysis by the deubiquitinating enzyme USP-7 (Figure 5a,c).

To further corroborate this result, we tested the processing of the Ub-p53-peptide conjugates by another deubiquitinating enzyme, namely ubiquitin C-terminal hydrolase-3 (UCHL-3). Ubiquitin C-terminal hydrolases are a family of cysteine hydrolases that catalyze the hydrolysis of amides, esters, and thioesters from the C-terminus of ubiquitin. These enzymes process ubiquitin precursors and ubiquitinated proteins to generate monomeric ubiquitin. However, the individual RP-HPLC profile of the above conjugates did not show any additional peaks, indicating that both conjugates were recalcitrant to cleavage by UCHL-3 (Figure 5b,d).

Both of the DUB enzymes (USP-7 and UCHL3) have been reported to recognize and cleave linear Ub-fusion proteins, Ub-precursors, and ubiquitinated products.^41^ In this study, they do recognize and cleave linear WT Ub with an extended C-terminal (Figure S7) but failed to recognize the Ub-p53-peptide conjugates with an engineered C-terminal (Figure 5). The engineering of C-terminal LRLRGG of Ub to LRLPXTGG does not involve mutation of the residues reportedly involved in interactions of Ub with ubiquitin binding domains (UBDs).^41,42^ Also, the L_73_, which confers resistance from DUBs activity when mutated to Pro, is also intact. The presence of Pro residue in the Ub C-terminal region alters its topology and may affect the formation of required hydrophobic interactions between neighboring Leu and active site residues of DUBs as reported earlier.^43^ Taken together, the above results establish the recalcitrant nature of these engineered Ub-p53-peptide conjugates toward hydrolysis by deubiquitinating enzymes. These results are in corroboration with the previous observation where engineered di-Ubs generated by sortase-mediated transpeptidation and genetic-code expansion were resistant to cleavage by the deubiquitinating enzyme USP2_CD_.^24^

## Discussion

The complexity of the ubiquitination system and its role in several physiological functions, especially proteasomal protein degradation, has been investigated for several decades now. The diversity in the modification site, Ub chain length, and intrachain lysine connectivity present tremendous challenges in the synthesis and purification of homogenously ubiquitinated proteins with native isopeptide.^44^ However, the development of genetic engineering methods and recent breakthrough in site-selective and site-specific conjugation methods has greatly helped in the preparation of site-specific polyUb chains and ubiquitinated POI for in vitro investigation of protein ubiquitination and deubiquitination.^45^ With the discovery of various ligases, such as sortase, OaAEP, butelase, Ubc9, etc., the chemoenzymatic synthesis of these ubiquitinated proteins is also gaining attention.

The use of SML strategies for generating protein conjugates having a native isopeptide bond has also been reported earlier, but in almost all of these strategies, class A sortases have been used, especially SaSrtA. Earlier, the in vitro semisynthesis of defined SUMO conjugates using SaSrtA was achieved by incorporating the sortase recognition motif LPQTG in the C-terminal region of SUMO proteins.^26^ Ubc9, an E2-SUMO-conjugating enzyme, has also been used for in vitro conjugation of wildtype Ub and ISG15 to recombinant proteins.^27^ A couple of combinatorial strategies using genetic-code expansion in combination with either SaSrtA or AEPs were also developed to prepare site-specific Ub and SUMO conjugates in vivo in an inducible fashion.^24,28^ However, genetic-code expansion is a difficult process and requires optimized systems for the generation of the orthogonal aminoacyl-tRNA synthetase/tRNA pair and incorporation of unnatural amino acids (UAAs) in living cells, whereas in vitro enzymatic generation of site-specific Ub conjugates is less time consuming and relatively easy to perform.

In this work, we have efficiently used two different housekeeping sortases, SaSrtA and SavSrtE, from class A and class E, respectively, for in vitro synthesis of defined Ub-p53 peptide conjugates. To the best of our knowledge, this is the first instance where a class E sortase, SavSrtE, alone or in combination with other sortase has been used for synthesizing protein–protein conjugates linked via a native isopeptide bond. SavSrtE is a Ca^2+^-independent sortase and recognizes both LAXTG and LPXTG pentapeptide motifs as its substrate.^35^ This substrate specificity for LPXTG recognition motif makes SavSrtE an attractive tool for this comparative study with SaSrtA.

Ubiquitin is conjugated via an isopeptide bond to the ε-NH_2_ of the Lys residue in substrate proteins through its C-terminal Gly residue.^1^ Although Ub exhibits a β-grasp fold containing a characteristic “ββαββαβ” core structure, the five C-terminal residues (RLRGG) are highly flexible and unstructured.^46^ So, engineering these corresponding residues (L_71_RLRGG_76_) to generate the sortase recognition motif LRL(PNT/PET)GG will keep its native structure intact. It will also keep the two most important residues L_71_ and L_73_ in the C-terminal region intact for the formation of a hydrophobic patch around I_36_, which is obligatory for recognition by some UBDs.^41^ However, L_71_ of Ub is engaged in the compact C-terminal β-grasp fold of Ub, so it may restrict the access of SaSrtA/SavSrtE to the sortase recognition pentapeptide motif at the C-terminus of Ub because the total pentapeptide sortase recognition motif is recognized efficiently preferably in unstructured form. Hence, the LRLPXTGG sortase recognition motif was generated by engineering a PXT substitution/insertion for R_74_ in the Ub C-terminal L_71_RLR_74_GG_76_. The engineered ubiquitins were purified and conjugated to GGGKY and di-Gly appended p53 ubiquitin target sequence, i.e., LMFK(ε-GG)TEG, successfully in the presence of SaSrtA/SavSrtE by SML.

Sortase-generated Ub-p53-peptide conjugates display a native isopeptide bond between the C-terminal glycine of Ub and the di-Gly appended p53-peptide target sequence. Hence, the structural aspects of these conjugates are almost identical to those of the natural Ub conjugates. Also, the most important residues, such as I_44_, F_4_, D_58_, TEK box, and L_71_ and L_73_ (C-terminus tail), which are involved in interactions of Ub with UBDs are preserved.^41^ Still, the newly synthesized Ub-LRLPNTLMFK(ε-GG)TEG and Ub-LRLPETLMFK(ε-GG)TEG conjugates were found to be resistant to hydrolysis by the deubiquitinating enzymes UCHL-3 and USP-7. This presumably could happen due to the altered sequence changes (presence of two extra residues at the C-terminus) or conformational changes occurring because of the presence of the Pro residue.^43^ These results are further supported by the findings of the Lang and co-workers, as the isopeptide bond of di-Ubs generated by sortase-mediated transpeptidation and genetic-code expansion are also resistant to cleavage by the deubiquitinating enzyme USP2_CD_.^24^

In this work, we propose an in vitro sortase-mediated chemoenzymatic strategy for semisynthesis of DUB-resistant site-specific Ub-peptide conjugates with a native isopeptide bond. For this purpose, we have resourcefully used two different housekeeping sortases, modified Ubs with an LPXTGG motif at their C-terminus and the di-Gly appended p53 peptide. The resultant Ub conjugates are also recalcitrant to DUB hydrolysis. This is a simple in vitro strategy which can be performed with ease in any biology laboratory with minimum requirements. This is also the first report of using a class E sortase as a protein engineering tool. Further research is required to take full advantage of the orthogonal specificity and Ca^2+^-independent nature of SavSrtE in protein engineering applications. It will also be interesting to combine the current strategy with engineered Ub-LAXTG SavSrtE recognition motif for developing in cellulo Ca^2+^-independent sortylation applications. This simple one-step sortase-based chemoenzymatic ligation strategy provides a route for the facile semisynthesis of homogeneous and defined isopeptide-linked ubiquitin-polypeptide conjugates and will help in understanding the complexity of the ubiquitination machinery. This strategy can be further extended to generate isopeptide-linked full protein-ubiquitin conjugates, which would further pave the way for designing peptide-based drugs and therapeutics for neurological disorders. The possibility of introducing different types of PTMs on a single protein by a simple and effective sortase-based strategy could allow us to probe the effect of combinatorial PTMs events in the field of intracellular signaling and other cellular functions. Although the generation of full protein-ubiquitin conjugates is more complicated both technically and experimentally, as it will require the incorporation of K(epsilon-GG) into a native protein, but it can be achieved in vitro using available strategies like SPPS and enzymatic ligation in combination with native chemical ligation, click chemistry, etc. Furthermore, the efficiency of chemoenzymatically modifying an intact protein may differ from the pentapeptide modification efficiency. In addition to this, the modification of surface lysine in an intact protein may have effects on its accessibility to the enzyme’s active site. These issues still need to be answered and require further dedicated investigation to realize the chemoenzymatic synthesis of site-specifically modified full length native protein.

## Supporting Information Available

Tables S1–S5: sequence alignment of ubiquitin (Ub) protein of various organisms, molecular masses of proteins and peptides, and list of primers; and Figures S1–S7: SDS gel profile of purified SavSrtE and SaSrtA enzymes, RP-HPLC chromatograms of the peptide and protein reactions, and structure of GGGKY peptide (PDF)
