# Supplementary material for: One-Step Sortase-Mediated Chemoenzymatic Semisynthesis of Deubiquitinase-Resistant Ub-Peptide Conjugates
Source: ACS Omega. 2022 Dec 7;7(50):46693–701. doi: 10.1021/acsomega.2c05652 (PMC9773336; doi:10.1021/acsomega.2c05652)
Supplement: Supplementary file 1 — ao2c05652_si_001.pdf [file ao2c05652_si_001.pdf]

Supporting information

One-step sortase-mediated chemoenzymatic semisynthesis of deubiquitinase-resistant Ub-peptide conjugates

Avinash K. Singh[a,b*], Sumit Murmu[b], Artur Krężel[a]

[a]*Department of Chemical Biology, Faculty of Biotechnology, University of Wrocław, F. Joliot-Curie 14a, 50-383 Wrocław, Poland*

[b]*National Institute of Immunology, Aruna Asaf Ali Marg, New Delhi 110067, India.*

*corresponding author, e-mail: Dr. Avinash K. Singh, avinashkumar.singh@uwr.edu.pl

Keywords: protein engineering, isopeptide bond, peptide ligation, solid-phase peptide synthesis, post-translational modification, sortase

Table S1. Sequence alignment of ubiquitin (Ub) protein of various organisms. Black color represents identical residues, blue color represents conserved residues and red color denotes unconserved residues. Bold highlights the LRLRGG sequence at the C-terminal tail utilized in this study.

Organism	Amino acid sequence
Oat	MQIFVKTLTGKTITLEVESSDTIDNVKAKIQDKEGIPPDQQRLIFAGKQLEDGRTLADYNIQKESTLHLV**LRLRGG**
Diamondback moth	MQIFVKTLTGKTITLEVEASDTIENVKAKIQDKEGIPPDQQRLIFAGKQLEDGRTLSDYNIQKESTLHLV**LRLRGG**
American cockroach	MQIFVKTLTGKTITLEVEASDTIENVKAKIQDKEGIPPDQQRLIFAGKQLEDGRTLSDYNIQKESTLHLV**LRLRGG**
M. bacterium	MQIFVKTLTGKTITLEVEPSDTIDNVKQKIQDKEGIPPDQQRLIFAGKQLEDGRTLSDYNIQKESTLHLV**LRLRGG**
Tomato	MQIFVKTLTGKTITLEVESSDTIDNVKAKIQDKEGIPPDQQRLIFAGKQLEDGRTLADYNIQKESTLHLV**LRLRGG**
F. cylindrus	MQIFVKTLTGKTITLDVESSDTIDNVKTKIQDKEGIPPDQQRLIFAGKQLEDGRTLSDYNIQKESTLHLV**LRLRGG**
Lesser duckweed	MQIFVKTLTGKTITLEVESSDTIDNVKAKIQDKEGIPPDQQRLIFAGKQLEDGRTLSDYNIQKESTLHLV**LRLRGG**
P. cylindrospora	MQIFVKTLTGKTITLEVESSDTIDNVKAKIQDKEGIPPDQQRLIFAGKQLEDGRTLSDYNIQKESTLHLV**LRLRGG**
E. fungus	MQIFVKTLTGKTITLEVESSDTIDNVKAKIQDKEGIPPDQQRLIFAGKQLEDGRTLGDYNIQKESTLHLV**LRLRGG**
Arabian camel	MQIFVKTLTGKTITLEVEPSDTIENVKAKIQDKEGIPPDQQRLIFAGKQLEDGRTLSDYNIQKESTLHLV**LRLRGG**
Aloe vera	MQIFVKTLTGKTITLEVESSDTIDNVKAKIQDKEGIPPDQQRLIFAGKQLEDGRTLADYNIQKESTLHLV**LRLRGG**
House fly	MQIFVKTLTGKTITLEVEPSDTIENVKAKIQDKEGIPPDQQRLIFAGKQLEDGRTLSDYNIQRESTLHLV**LRLRGG**
Amoeba	MQIFVKTLTGKTITLEVESSDTIENVKQKIQDKEGIPPDQQRLIFAGKQLEDGRTLADYNIQKESTLHLV**LRLRGG**
Baker's yeast	MQIFVKTLTGKTITLEVESSDTIDNVKSKIQDKEGIPPDQQRLIFAGKQLEDGRTLSDYNIQKESTLHLV**LRLRGG**
Rice	MQIFVKTLTGKTITLEVESSDTIDNVKAKIQDKEGIPPDQQRLIFAGKQLEDGRTLADYNIQKESTLHLV**LRLRGG**
C. elegans	MQIFVKTLTGKTITLEVEASDTIENVKAKIQDKEGIPPDQQRLIFAGKQLEDGRTLSDYNIQKESTLHLV**LRLRGG**
Red alga	MQIFVKTLTGKTITLEVESSDTIENVKTKIQDKEGIPPDQQRLIFAGKQLEDGRTLSDYNIQKESTLHLV**LRLRGG**
Purple sea urchin	MQIFVKTLTGKTITLEVEPSDSIENVKAKIQDKEGIPPDQQRLIFAGKQLEDGRTLSDYNIQKESTLHLV**LRLRGG**
Garden pea	MQIFVKTLTGKTITLEVESSDTIDNVKAKIQDKEGIPPDQQRLIFAGKQLEDGRTLADYNIQKESTLHLV**LRLRGG**
Mouse	MQIFVKTLTGKTITLEVEPSDTIENVKAKIQDKEGIPPDQQRLIFAGKQLEDGRTLSDYNIQKESTLHLV**LRLRGG**
Human	MQIFVKTLTGKTITLEVEPSDTIENVKAKIQDKEGIPPDQQRLIFAGKQLEDGRTLSDYNIQKESTLHLV**LRLRGG**

Table S2. Expected and observed molecular masses of ubiquitin, Ub mutants, and sortases.

Ub protein	Mass mono. expected (Da)	Mass mono. observed (Da)
WT Ub-LRLRGGLEH$_6$	9624.10	9623.75
Ub-LPLTGGLEH$_6$	9509.99	9509.1
Ub-LPNTGGLEH$_6$	9510.95	9509.98
Ub-LRLPNTGGLEH$_6$	9780.14	9780.45
Ub-LRLPETGGLEH$_6$	9795.14	9790.49
Ub-LRLPLTGGLEH$_6$	9779.18	9777.22
SaSrtA Δ59	17865.0	17864.7
SavSrtE Δ50	22289.0	22288.0

Table S3. Expected and observed molecular masses of peptides synthesized in this study.

Peptide	Mass mono. expected (Da)	Mass mono. observed (Da)
GGGKY	480.23	480.2004
LMFK(ε-GG)TEG	938.45	937.53

Table S4. Expected and observed molecular masses of synthesized Ub conjugates.

Ub conjugate	Mass mono. expected (Da)	Mass mono. observed (Da)
Ub-LRLPNTGGGKY	9063.84	9063.31
Ub-LRLPETGGGKY	9078.84	9077.48
Ub-LRLPNTLMFK(ε-GG)TEG	9522.06	9521.55
Ub-LRLPETLMFK(ε-GG)TEG	9537.06	9539.32

Table S5. Sequences of primers used in this study.

**WT-Ub-LRLRGG**	
**Fwd**	5′-CCCCCCATATGCAGATCTTCGTGAAAACCCTTACCGG -3′
**Rev**	5′-CCCCCCTCGAGACCACCTCTCAGACGCAGGAC -3′
**Ub-LPLTGG**	
**Fwd**	5′ ACCCTGCACCTGGTCCTGCCGCTGACCGGTGGTCTCGAGCACCAC 3′
**Rev**	5′ GTGGTGCTCGAGACCACCGGTCAGCGGCAGGACCAGGTGCAGGGT 3′
**Ub-LPNTGG**	
**Fwd**	5′ ACCCTGCACCTGGTCCTGCCGAACACCGGTGGTCTCGAGCACCAC 3′
**Rev**	5′ GTGGTGCTCGAGACCACCGGTGTTCGGCAGGACCAGGTGCAGGGT 3′
**Ub-LRLPNTGG**	
**Fwd**	5′ CACCTGGTCCTGCGTCTGCCAAACACTGGTGGTCTCGAGCACCAC 3′
**Rev**	5′ GTGGTGCTCGAGACCACC**AGTGTTTGG**CAGACGCAGGACCAGGTG 3′
**Ub-LRLPETGG**	
**Fwd**	5′ CACCTGGTCCTGCGTCTG**CCAGAAACT**GGTGGTCTCGAGCACCAC 3′
**Rev**	5′ GTGGTGCTCGAGACCACC**AGTTTCTGG**CAGACGCAGGACCAGGTG 3′
**Ub-LRLPLTGG**	
**Fwd**	5′ CACCTGGTCCTGCGTCTG**CCACTGACT**GGTGGTCTCGAGCACCAC 3′
**Rev**	5′ GTGGTGCTCGAGACCACC**AGTCAGTGG**CAGACGCAGGACCAGGTG 3′

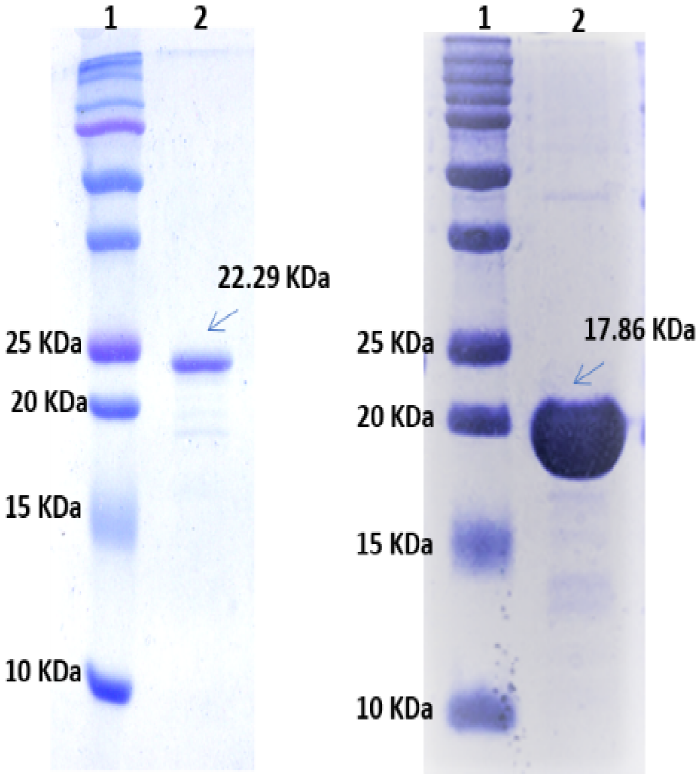

Figure S1. SDS gel profile of purified SavSrtE (left) and SaSrtA (right) enzymes. SavSrtE (MW 22289 Da) and SaSrtA (MW 17865 Da) were expressed and purified from *E. coli* by Ni-NTA affinity purification followed by desalting on PD-10 columns.

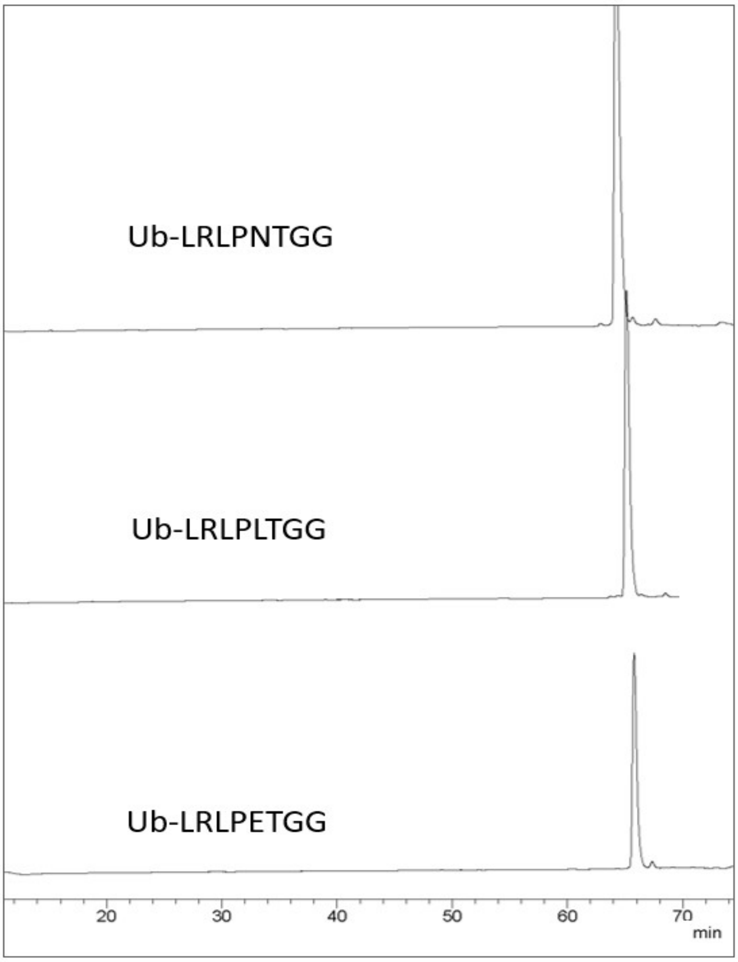

Figure S2. Exemplary RP-HPLC purification of Ub-LRLPNTGG (top), Ub-LRLPLTGG (middle), and Ub-LRLPETGG (bottom) on a C-18 column in a linear gradient of 8-72% ACN in 130 minutes and absorbance was recorded at 210 nm.

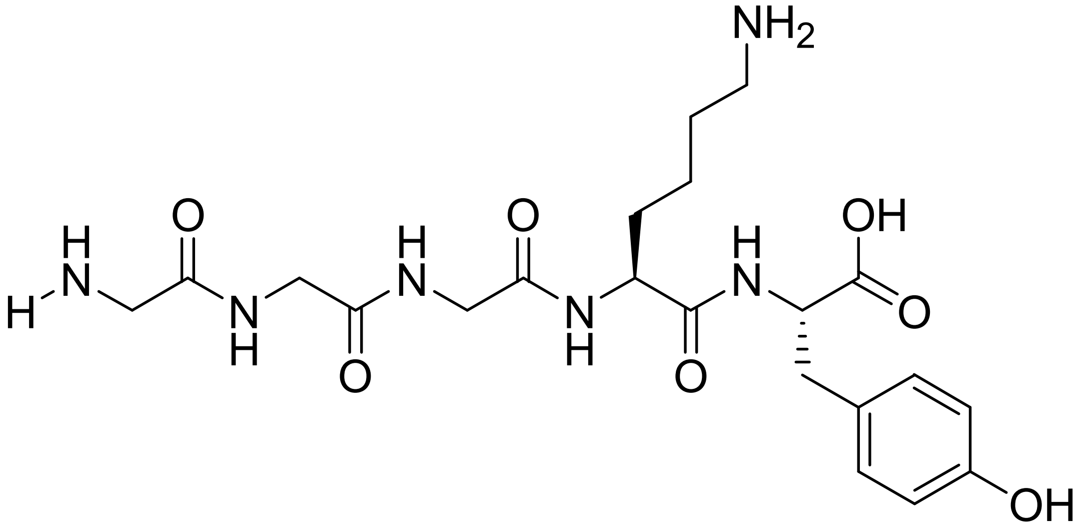

Figure S3. Structure of GGGKY peptide used for the sortase-mediated ligation with ubiquitin mutants generated for the semisynthesis of Ub conjugates.

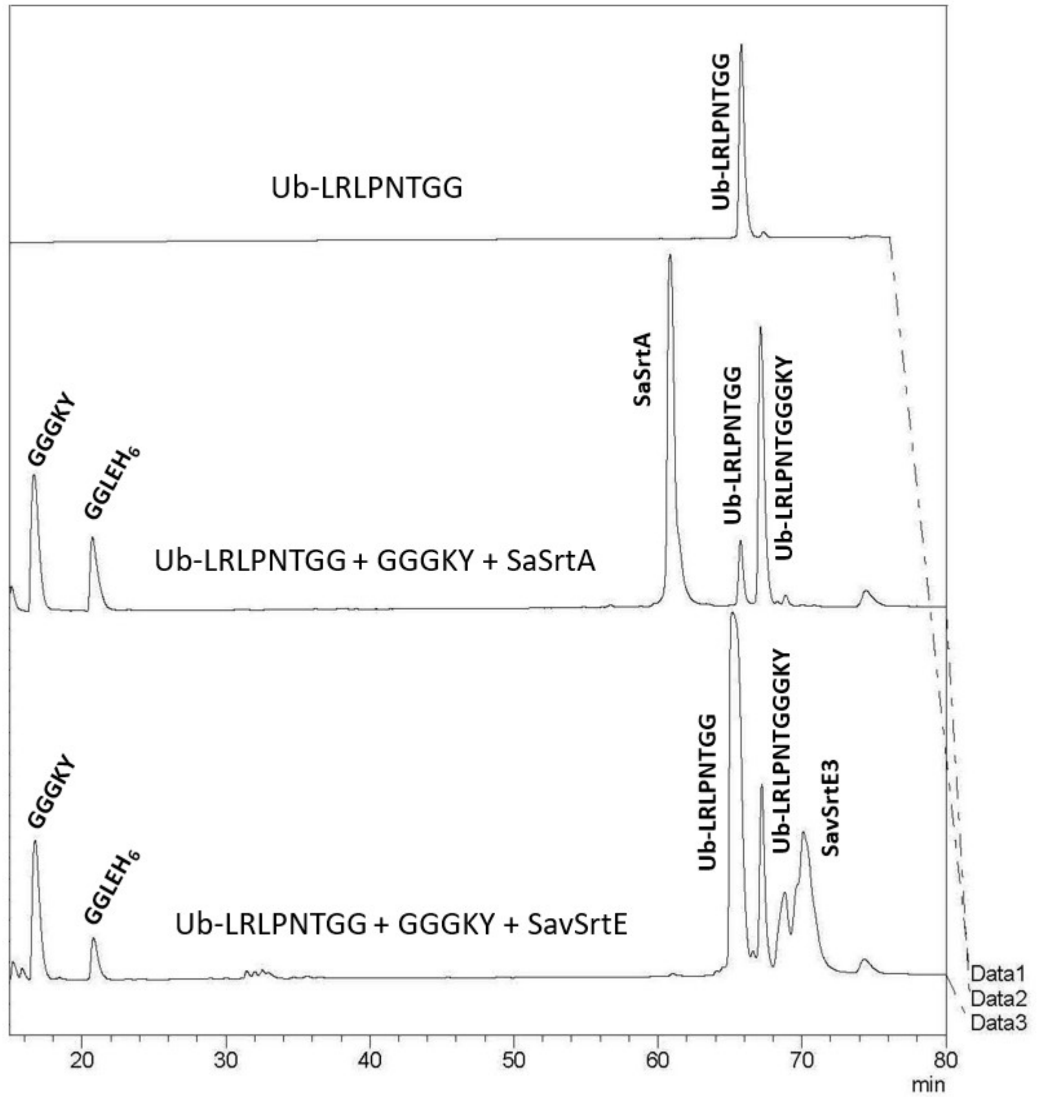

Figure S4. RP-HPLC reaction profiles of 0.5 mM Ub-LRLPNTGG control (top), transpeptidation reaction between 0.5 mM Ub-LRLPNTGG and 1 mM GGGKY with 50 µM SaSrtA (middle), and transpeptidation reaction between 0.5 mM Ub-LRLPNTGG and 1 mm GGGKY with 100 µM SavSrtE (bottom). Analysis was done on a C-18 column in a linear gradient of 8-72% ACN in 130 minutes and absorbance was recorded at 210 nm.

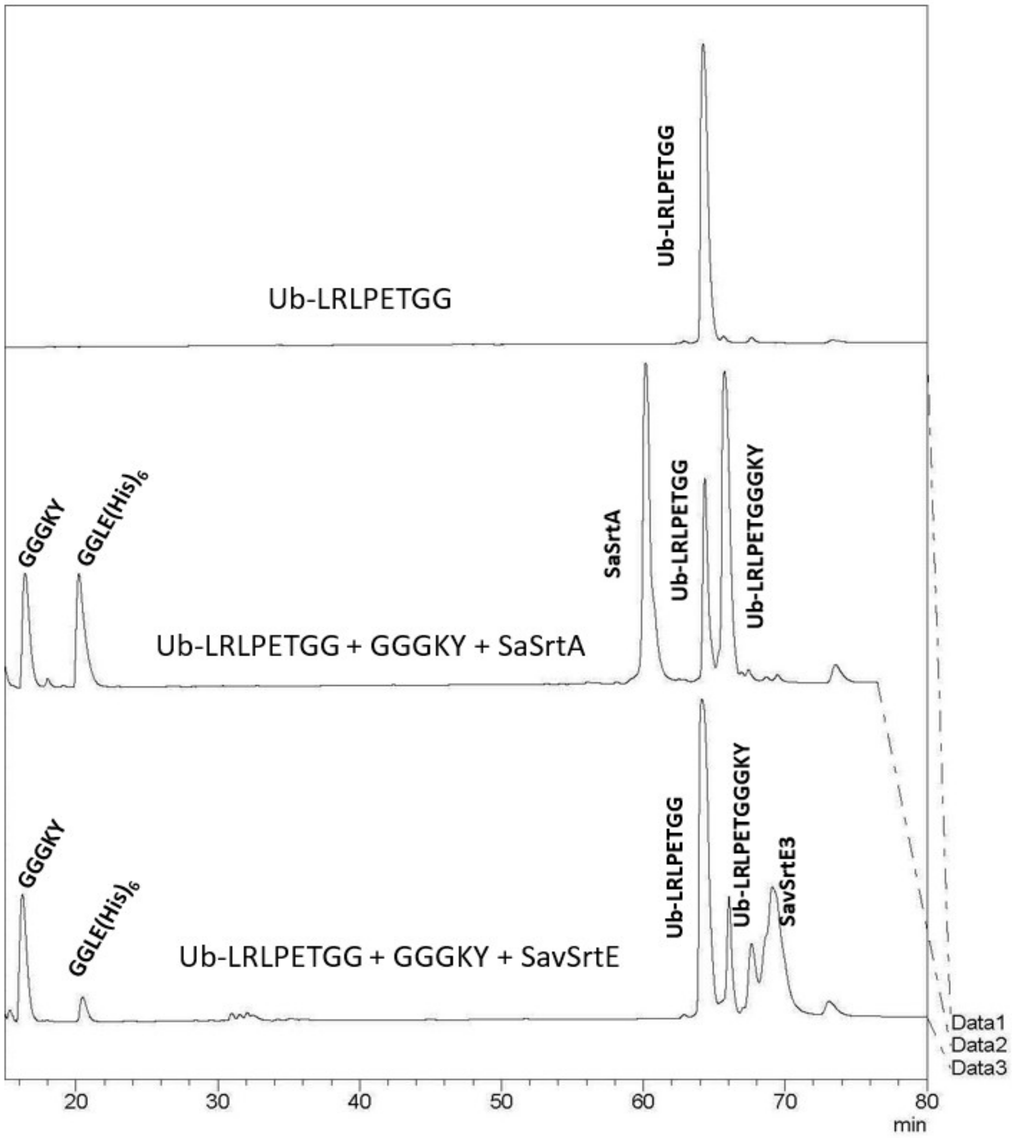

Figure S5. RP-HPLC reaction profiles of 0.5 mM Ub-LRLPETGG control (top), transpeptidation reaction between 0.5 mM Ub-LRLPETGG and 1 mM GGGKY with 50 µM SaSrtA (middle), and transpeptidation reaction between 0.5 mM Ub-LRLPETGG and 1 mM GGGKY with 100 µM SavSrtE (bottom). Analysis was done on a C-18 column in a linear gradient of 8-72% ACN in 130 minutes and absorbance was recorded at 210 nm.

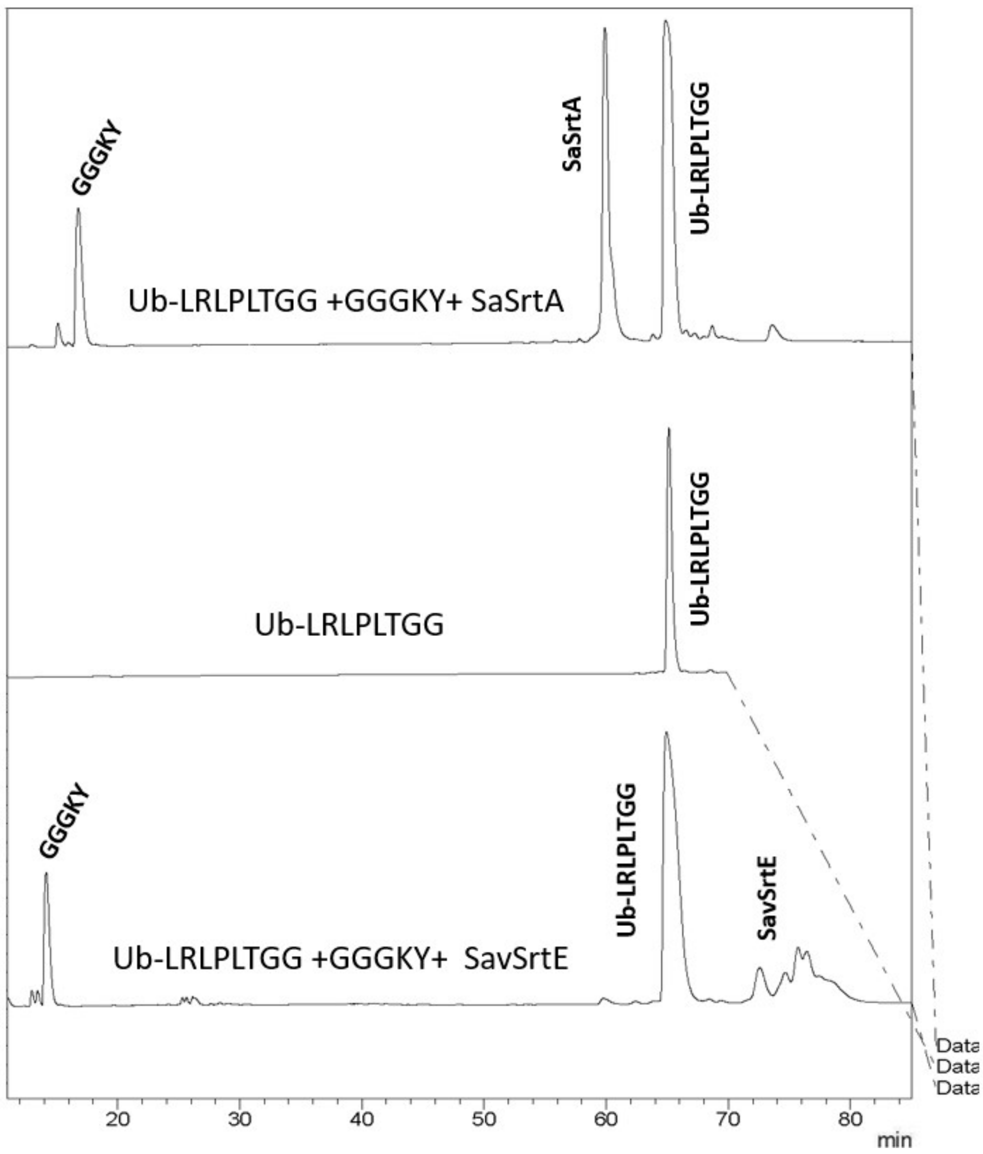

Figure S6. RP-HPLC reaction profiles of transpeptidation reaction between 0.5 mM Ub-LRLPLTGG and 1 mM GGGKY with 50 µM SaSrtA (top), 0.5 mM Ub-LRLPLTGG control (middle), and transpeptidation reaction between 0.5 mM Ub-LRLPLTGG and 1 mM GGGKY with 100 µM SavSrtE (bottom). Analysis was done on a C-18 column in a linear gradient of 8-72% ACN in 130 minutes and absorbance was recorded at 210 nm.

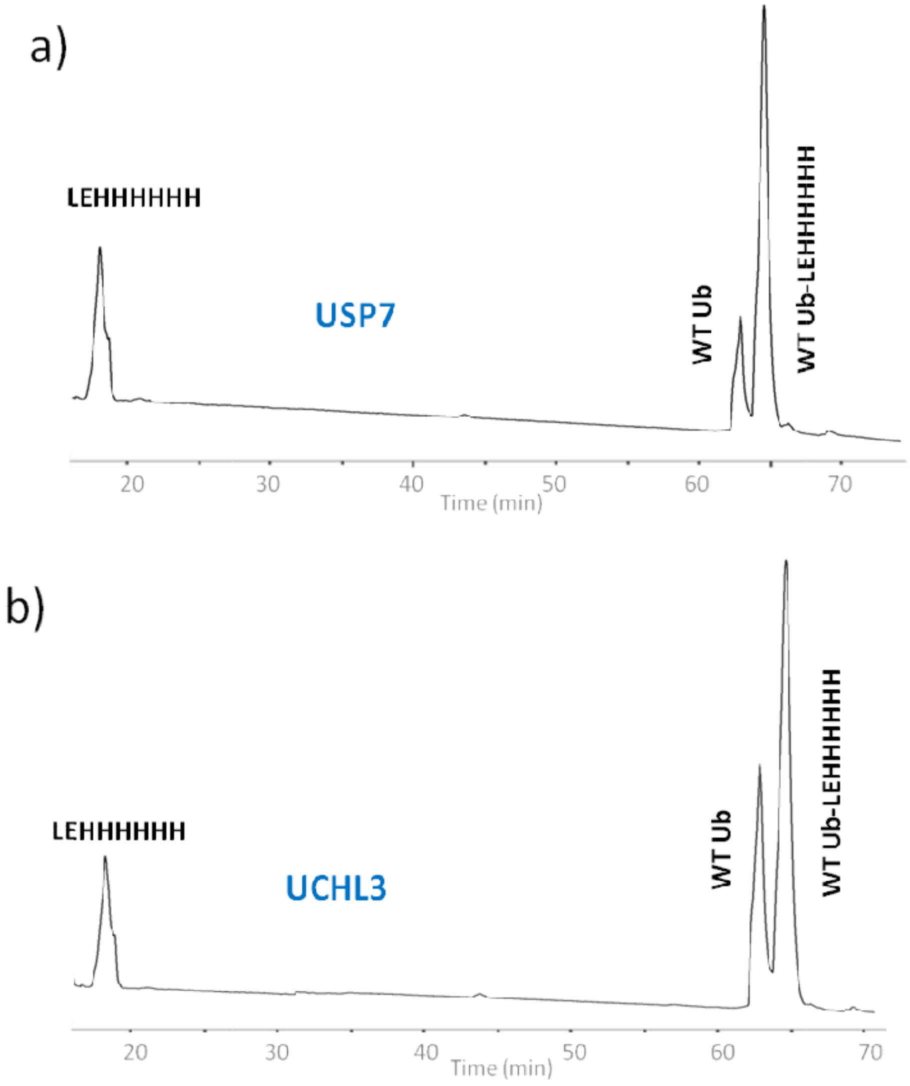

Figure S7. Hydrolysis of WT Ub-LEHHHHHH. 0.5 mM WT Ub-LEHHHHHH was incubated with 1 µM of DUB at 37°C for 1 h and was analyzed at 210 nm on a C-18 RP-HPLC column with a linear gradient of 4-72% ACN in 130 minutes. a) RP-HPLC analysis of the reaction between WT Ub-LEHHHHHH and USP7; b) RP-HPLC analysis of the reaction between WT Ub-LEHHHHHH and UCHL3.
